# Phenylephrine-Induced Cardiovascular Changes in the Anesthetized Mouse: An Integrated Assessment of *in vivo* Hemodynamics Under Conditions of Controlled Heart Rate

**DOI:** 10.3389/fphys.2022.831724

**Published:** 2022-02-17

**Authors:** Rajkumar Rajanathan, Tina Myhre Pedersen, Morten B. Thomsen, Hans Erik Botker, Vladimir V. Matchkov

**Affiliations:** ^1^Department of Biomedicine, Aarhus University, Aarhus, Denmark; ^2^Department of Biomedical Sciences, University of Copenhagen, Copenhagen, Denmark; ^3^Department of Cardiology, Aarhus University Hospital, Aarhus, Denmark

**Keywords:** cardiovascular function, phenylephrine, open-thorax, blood pressure, cardiac output, stroke volume, hemodynamic, electrical pacing

## Abstract

**Objective:**

Investigating the cardiovascular system is challenging due to its complex regulation by humoral and neuronal factors. Despite this complexity, many existing research methods are limited to the assessment of a few parameters leading to an incomplete characterization of cardiovascular function. Thus, we aim to establish a murine *in vivo* model for integrated assessment of the cardiovascular system under conditions of controlled heart rate. Utilizing this model, we assessed blood pressure, cardiac output, stroke volume, total peripheral resistance, and electrocardiogram (ECG).

**Hypothesis:**

We hypothesize that (i) our *in vivo* model can be utilized to investigate cardiac and vascular responses to pharmacological intervention with the α_1_-agonist phenylephrine, and (ii) we can study cardiovascular function during artificial pacing of the heart, modulating cardiac function without a direct vascular effect.

**Methods:**

We included 12 mice that were randomly assigned to either vehicle or phenylephrine intervention through intraperitoneal administration. Mice were anesthetized with isoflurane and intubated endotracheally for mechanical ventilation. We measured blood pressure *via* a solid-state catheter in the aortic arch, blood flow *via* a probe on the ascending aorta, and ECG from needle electrodes on the extremities. Right atrium was electrically paced at a frequency ranging from 10 to 11.3 Hz before and after either vehicle or phenylephrine administration.

**Results:**

Phenylephrine significantly increased blood pressure, stroke volume, and total peripheral resistance compared to the vehicle group. Moreover, heart rate was significantly decreased following phenylephrine administration. Pacing significantly decreased stroke volume and cardiac output both prior to and after drug administration. However, phenylephrine-induced changes in blood pressure and total peripheral resistance were maintained with increasing pacing frequencies compared to the vehicle group. Total peripheral resistance was not significantly altered with increasing pacing frequencies suggesting that the effect of phenylephrine is primarily of vascular origin.

**Conclusion:**

In conclusion, this *in vivo* murine model is capable of distinguishing between changes in peripheral vascular and cardiac functions. This study underlines the primary effect of phenylephrine on vascular function with secondary changes to cardiac function. Hence, this *in vivo* model is useful for the integrated assessment of the cardiovascular system.

## Background

Cardiovascular morbidity is the leading cause of mortality worldwide (Mc Namara et al., 2019). Understanding the molecular mechanisms underlying cardiovascular morbidity is, therefore, of utmost importance to provide new therapeutic insight and preventive strategies to reduce mortality and improve the quality of life for patients. Many great discoveries have been made through *in vivo* assessment of cardiovascular function. Animal models, particularly mice, are often used for this purpose as they are easily accessible and compatible with research on molecular mechanisms underlying cardiovascular physiology and pathology (Janssen et al., 2002; Zaragoza et al., 2011; Camacho et al., 2016). However, many of the suggested molecular mechanisms important for cardiovascular function are based on studies that are limited to isolated organ functions or basic phenotyping reports on tissue perfusion and blood pressure changes. Thus, a comprehensive cardiovascular functional phenotype is rarely described. Existing models have provided significant improvements to the therapeutic armamentarium against cardiovascular diseases, however, it remains the leading cause of death and disability worldwide (Pandya et al., 2013). Hence, new research modalities and an improvement of existing methods are essential to further progress our understanding of mechanisms underlying cardiovascular morbidity (Wier, 2014; Figtree et al., 2021).

Studying the cardiovascular system is a complex task. The cardiovascular system is a closed system consisting of the heart as a pump and blood vessels in which blood flows through. Thus, the efficiency of the circulation is determined by cardiac function, i.e., contractility and heart rate, in conjunction with vascular diameter changes determining total peripheral resistance (Mayet and Hughes, 2003). This interplay between cardiac and vascular functions is further complicated by tight regulation from numerous humoral factors and the autonomic nervous system under normal physiological conditions (Holmes et al., 2004; Gordan et al., 2015; Song et al., 2015; Paz Ocaranza et al., 2020). Evidently, there is a vast number of regulators of the cardiovascular system complicating the mechanistic assessment of the molecular background underlying cardiovascular function under normal physiological conditions. Apart from biological variation including aging and fitness level, deviation from normal physiological conditions due to pathology, e.g., changed sympathetic activity in hypertension, may further contribute to the complexity of the cardiovascular system (Kougias et al., 2010). All these factors must be considered when extrapolating *ex vivo* knowledge onto whole body homeostasis.

Most conventional ways to investigate the cardiovascular system lack the integrated assessment. For instance, cardiac function is extensively assessed *in vivo* through various imaging modalities, e.g., echocardiography and magnetic resonance imaging (Ram et al., 2011; Botnar and Makowski, 2012; Li et al., 2020). Albeit this provides great knowledge on cardiac function *in vivo*, the results from these studies are often seen in isolation and not in conjunction to any physiological changes in peripheral vascular resistance and blood pressure. Vascular function is routinely studied *ex vivo*, for instance, through different forms of myography, but this is a highly simplified approach as it does not permit simultaneous assessment of changes in blood flow and transmural pressure that take place *in vivo* (Mulvany and Aalkjaer, 1990). Perfusion of the hind limb or another vascular bed provides a good insight into changes in the peripheral resistance, but this procedure can only be done under conditions of either constant flow or constant pressure (Bomzon and Naidu, 1985). Many studies conclude on vascular function based on blood pressure measurements (Zhao et al., 2011) or tissue perfusion assessment with imaging techniques (Moroz and Reinsberg, 2018), but the conclusions are limited due to lacking information on cardiac output or volume blood flow and driving perfusion pressure, respectively, which are key components for tissue perfusion. Hence, the conventional models are unable to properly address current pressing issues in cardiovascular physiology. One of the challenges is elucidating the mechanisms of molecular signaling or a pathological state with multifarious cardiovascular effects (Tisdale and Gheorghiade, 1992; Holmes et al., 2004; Song et al., 2015; Nielsen et al., 2019; Paz Ocaranza et al., 2020). Moreover, the concurrent assessment of load-dependent and independent parameters is valuable in all disease states and after drug administration. This can be achieved in an integrated model that makes the separation of intrinsic cardiac parameters and vascular responses feasible.

Consequently, the field of cardiovascular physiology demands a comprehensive model for the integrated assessment of the cardiac and vascular functions and their responses to pharmacological intervention. Considering this, we propose an experimental protocol for the integrated assessment of hemodynamic parameters of anesthetized mice before and after pharmacological intervention. By fully elucidating the cardiovascular mechanisms, including load-dependent and independent parameters, our method may contribute to providing valuable new therapeutic targets. In this study, we have challenged mice with a selective α_1_ adrenoreceptor agonist, phenylephrine, which has been described to affect both cardiac and vascular functions *in vivo* (Crosley et al., 1951; Malhotra et al., 1998). We aimed to validate our experimental protocol by distinguishing between phenylephrine-induced vascular and subsequent cardiac changes. We hypothesize that artificial pacing of the heart controls the cardiac function and, hence, can be helpful to differentiate between vascular and cardiac responses. The hemodynamic assessment includes blood pressure, cardiac output, stroke volume, total peripheral resistance, and electrophysiological parameters derived from the electrocardiogram (ECG), and their changes to increasing pacing frequencies.

## Materials and Methods

### Animals

Male mice (C57BL/6, Janvier Labs, France) at the age of approximately 9 months with a mean weight of approximately 33 g were used in this study. Mice were housed in rooms with temperature (21.5°C) and humidity (55%) regulation and with a 12:12 h light-dark cycle. Animals had access to *ad libitum* food and tap water. All animal experiments conformed to the guidelines from Directive 2010/63/EU of the European Parliament on the protection of animals used for scientific purposes. The experimental protocol was approved by the Animal Experiments Inspectorate of the Danish Ministry of Environment and Food and reported in accordance with the ARRIVE (Animal Research: Reporting *in vivo* Experiments) guidelines. Mice were euthanized immediately at the end of protocol under deep anesthesia by cervical dislocation.

### Anesthesia and Mechanical Ventilation

Anesthesia of mice was induced with 3% isoflurane mixed with 100% O_2_ for 5 min. Mice were then placed on a homeothermic blanket system (50-7222F, Harvard Apparatus, United States) to control core temperature (37 ± 5°C) and anesthesia was adjusted and maintained at 2% of isoflurane throughout. Hair was removed from the thorax and cervical regions of the supine mice with a depilatory agent (Veet, Canada). A microscope was used for the surgical procedures. Following a midline cervical incision, the trachea was visualized, and endotracheal intubation was performed. The intubation tube was connected to a MiniVent Ventilator (model 845, Harvard Apparatus, United States). The expired air was connected to a capnograph (Type 340, Harvard Apparatus, United States) reporting the end-tidal CO_2_ (% etCO_2_). Furthermore, positive end-expiratory pressure of 2 cm H_2_O was attained with a water lock consisting of a water-filled tube (Schwarte et al., 2000). An actuator head was connected to the inspiratory air circuit monitoring airway pressure and flow, and this was set to a maximum threshold of 15 cm H_2_O to prevent pulmonal barotrauma (Wilson et al., 2012). Tidal volume was kept constant at 10 μl/g body mass (Hauber et al., 2010). Ventilation rate was adjusted based on % etCO_2_ at approximately 3.5% (Hansen et al., 2020). Next, pancuronium-bromide (0.4 mg/h i.p., P1918, Sigma, United States), an M_2_-acetylcholine receptor blocker, was used to immobilize the mouse for the rest of protocol duration (Uhlirova et al., 2016), and 0.9% NaCl solution (1 ml i.p., 9 mg/ml NaCl, Fresenius Kabi, Germany) was provided to balance out any fluid loss.

### Arterial Blood Pressure Measurements

A 1.0 F solid-state catheter (SPR-1000, Millar, United States) connected to a bioamplifier unit (ADInstruments, Australia) was introduced into the common carotid artery and placed in the aortic arc for continuous blood pressure measurements. The right carotid artery was visualized and bluntly dissected using forceps. Special precaution was taken to prevent damage of the vagal nerve. To prevent the artery from drying out, it was kept wet during the implantation procedure with warm 0.9% NaCl saline. Three silk sutures (Multifilament 5-0, Teleflex Medical, United States) were used for cannulation and fixation of the catheters. The first suture was tightened in a double knot around the artery to occlude it as far cranially as possible. The second suture was placed under the artery and pulled caudally to temporarily occlude the artery. The third suture was prepared as a loose knot in the caudal end of the artery segment. The tip of a needle (25G × 5/8”, 0.5 mm × 16 mm, BD Microlance, United States) was bent in a 90° angle and used to puncture the carotid artery. The solid-state catheter was then introduced in the caudal direction through the puncture, and it was secured by tightening of the third suture. Once secured, the second suture was removed, and the catheter tip was advanced and placed in the aortic arc. The position of the catheter tip was confirmed by evaluations of the blood pressure pulse profile corresponding to blood pressure in aorta, and its location was also anatomically verified at the end of the experiment following dissection. To prevent loss of fluid through evaporation, the surgical area was covered with gauze wet with warm 0.9% NaCl saline.

### Transit-Time Flow Probe

A transit-time flow probe (1.5 SL, Transonic, United States) was mounted on the ascending aorta assessing the blood flow as surrogate measure for cardiac output (Janssen et al., 2002). Following a midsternal longitudinal incision exposing tissue on the right thoracic side, the mouse was repositioned on the left side exposing the right side of thorax to access the heart and the ascending aorta as described previously (Tarnavski et al., 2004). A longitudinal incision was made spanning from the first to third intercostal space 1–2 mm laterally from the sternal midline. The thoracotomy was expanded to approximately 1 cm in width using a small mouse retractor. The right anterior surface of the heart and ascending aorta were identified, and pericardiectomy was carefully performed. In case of thymus covering the ascending aorta, a partial thymectomy was performed. A blunt vascular hook (0.3 mm) and forceps were used for final blunt dissection and isolation of the ascending aorta. The transit-time flow probe was prepared by soaking it in ultrasound gel (Kruuse, Denmark) until signal quality was optimal. Once prepared, the flow probe was positioned on the ascending aorta. Successful placement of the flow probe was evident from stable representable flow recordings indicating no aortic compression. Cardiac output was calculated as the integral of the transit-time flow probe measurements.

### Electrical Pacing and Electrocardiogram Recording

**FIGURE 1:**
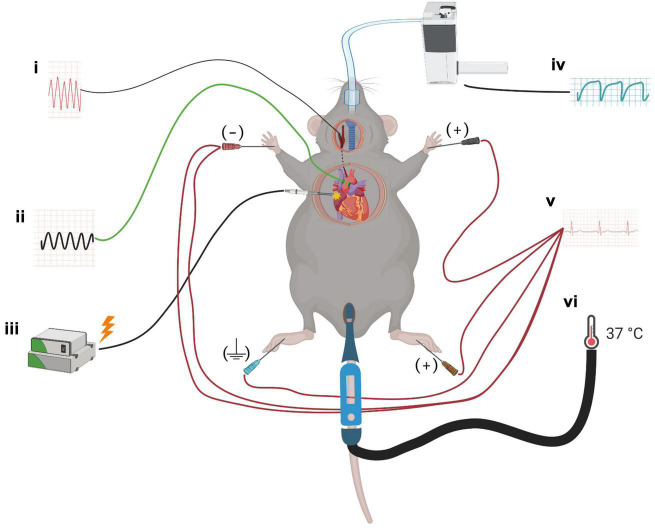
A schematic for the experimental setup. Mice were anesthetized with isoflurane, and probes to monitor the cardiovascular parameters were implanted: (i) solid-state pressure catheter was inserted through the common carotid artery and placed in the arcus aorta, (ii) transit-time flow probe was mounted on the ascending aorta to measure cardiac output, (iii) bipolar electrodes were placed at the right atrium for cardiac pacing, (iv) mice were intubated endotracheally for mechanical ventilation, (v) ECG needle electrodes were placed on all four limbs to monitor heart rate and ECG parameters, and (vi) throughout the surgery and protocol, mice were placed on a homeothermic blanket system with a rectal probe to maintain core temperature.

Platinum bipolar electrodes connected to a dual bioamplifier/stimulator unit (ADInstruments, Australia) were positioned on the right atrium in the area of the sinus node for electrical pacing of the heart. The electrical pulse width was 0.2 ms, and the current was 3 mA. ECG electrodes (MLA2505, ADInstruments, Australia) connected to a shielded 5-lead bioamplifier cable (MLA2540, ADInstruments, Australia) were placed on each limb for the recording of leads that correspond to lead I and II in humans as shown in Figure 1. The bioamplifier unit for the ECG recording was set to detect signals within a range of 10 mV with a low-pass filter (500 Hz), a high-pass filter (0.3 Hz), and a mains filter activated.

### Experimental Protocol

The experimental protocol consisted of two electrical pacing sessions with pharmacological intervention in between them. After ensuring steady baseline recordings of blood pressure, blood flow, and ECG parameters, the first pacing session was commenced. For each pacing session, the heart was initially paced at 10 Hz with stepwise increase to 10.3, 10.6, 11, and 11.3 Hz. Each frequency was maintained for a period of 20 s. Following a pause of 1 min an injection of either phenylephrine (0.3 mg/kg i.p., P6126, Sigma, United States) or a corresponding volume of 0.9% NaCl was given, with animals being allocated to each group according to a random number generator. The second pacing session was initiated 10 min after the injection. Data was collected for 6 min following the end of the second pacing session. Data were acquired using LabChart Pro 8 (ADInstruments, Australia) with a sampling rate of 1 k/s. Hemodynamic and electrophysiological parameters were averaged over a period of 20 s.

### Statistics

Data were analyzed using Prism 9 (GraphPad, United States) and presented as means ± SEM. Baseline values between the two groups were compared using unpaired *t*-test. The cardiovascular parameters obtained during pacing protocols and following pharmacological intervention were compared using two-way ANOVA with Bonferroni correction. Results were considered statistically significant at *P* < 0.05.

## Results

Instrumentation of blood pressure and flow probes was successful in all six mice, however, ECG data were only available for *n* = 5. The protocol was completed in all mice. Baseline parameters in the two groups of mice were comparable (Table 1).

**TABLE 1:** Bodyweight, age, and baseline ventilatory, and cardiovascular parameters of anesthetized mice prior to administration of either phenylephrine (0.3 mg/kg, i.p.) or vehicle, and prior to cardiac pacing.

Parameters	Vehicle group	Phenylephrine group	*P*
Body weight, g	33.2 ± 0.6	32.1 ± 0.8	0.31
Age, weeks	38.7 ± 1.2	37.3 ± 0.9	0.40
Systolic blood pressure, mmHg	73.2 ± 5.2	69.3 ± 4.8	0.44
Diastolic blood pressure, mmHg	46.1 ± 4.8	41.1 ± 3.9	0.58
Heart rate, BPM	537 ± 19	542 ± 7	0.79
Stroke volume, μl	16.4 ± 1.9	15.6 ± 1.8	0.78
Cardiac output, ml/min	8.8 ± 1.0	8.5 ± 0.9	0.83
Peripheral resistance, mmHg⋅min/ml	6.5 ± 0.5	6.2 ± 0.5	0.72
End-tidal CO_2_, %	3.4 ± 0.1	3.3 ± 0.2	0.68
Ventilation rate, min^–1^	81.3 ± 2.9	87.0 ± 2.2	0.15

*Data were compared with unpaired t-test with n = 6 in both groups.*

### Electrical Pacing and Intervention With α_1_ Adrenergic Agonist Did Not Significantly Alter the Electrocardiogram or Cause Arrhythmia

**FIGURE 2:**
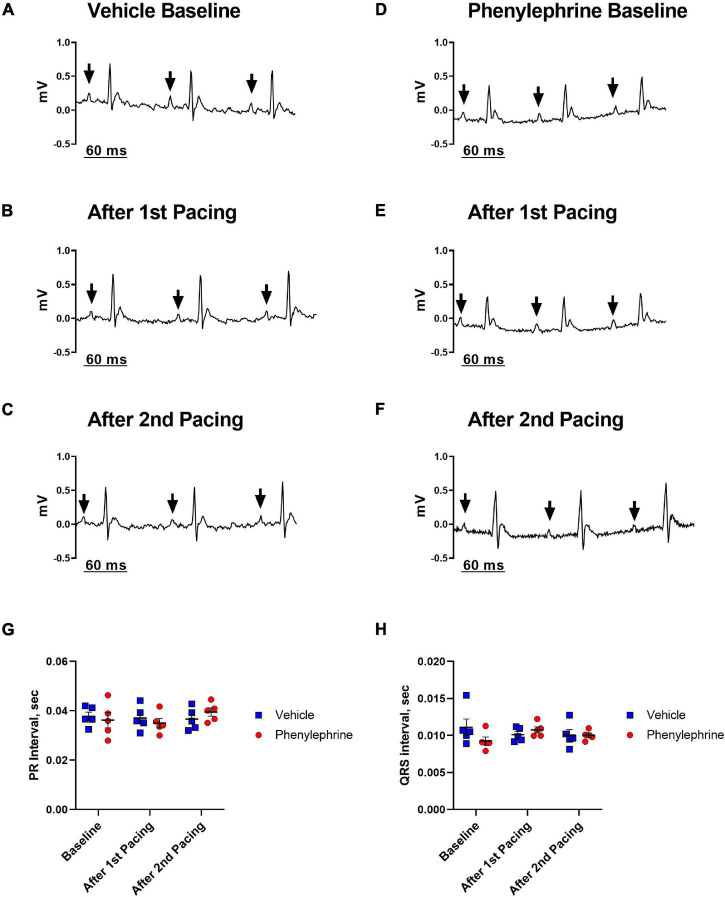
Representative electrocardiograms (lead I) recorded prior to and after both the first and second pacing sessions for the vehicle **(A–C)** and phenylephrine groups **(D–F)**. Black arrows indicate P waves with consecutive QRS complexes, thus, describing sinus rhythm before and after electrical pacing of the heart. **(G)** There were no differences between the mean PR interval between vehicle and phenylephrine groups before and after both pacing sessions (*P* = 0.92). **(H)** No differences in width of QRS complex were seen between the vehicle and phenylephrine groups (*P* = 0.47). Data were compared with two-way ANOVA, *n* = 5.

Electrical pacing of the right atrium did not interrupt atrioventricular conduction (Figures 2A–F). In both the first and second pacing sessions, PR interval and width of QRS complex were not significantly affected (Figures 2G,H). Furthermore, baseline values for the length of the PR interval and width of the QRS complex were similar between the phenylephrine and vehicle groups. Similarly, no differences were seen between the phenylephrine and vehicle groups in PR interval and width of QRS complex in between and after pacing sessions (Figures 2G,H).

### α_1_ Adrenergic Stimulation Significantly Elevated Blood Pressure but Not Cardiac Output Through Changes in Total Peripheral Resistance

**FIGURE 3:**
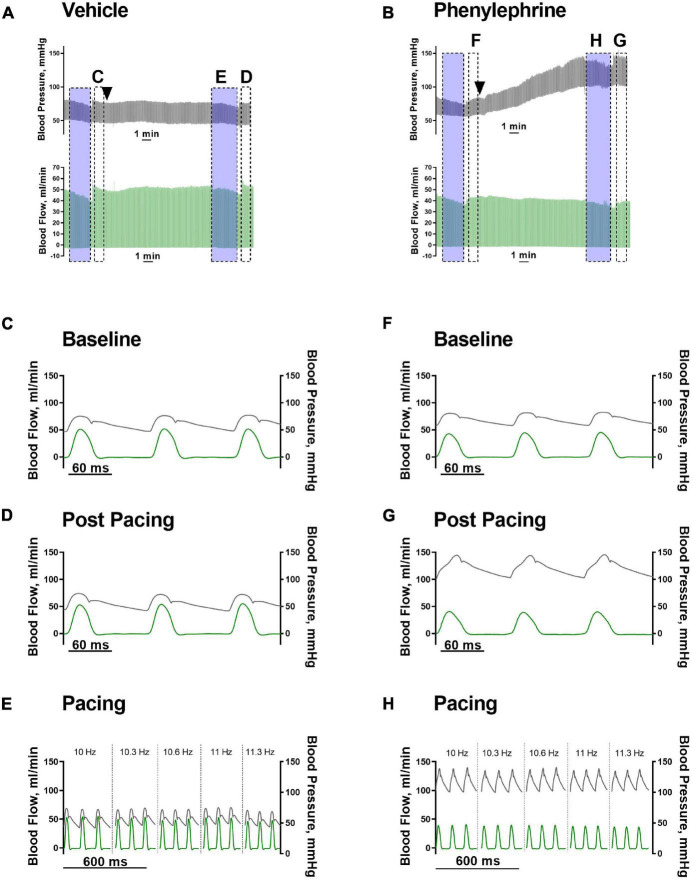
**(A,B)** Representative traces of blood pressure (black traces) and blood flow (green traces) after vehicle or phenylephrine administration (black arrow heads). Transparent blue boxes indicate periods of pacing. Panels **(C–H)** are indicated in panels **(A,B)**. **(C,F)** Hemodynamic parameters prior to vehicle or phenylephrine administration. **(D,G)** Hemodynamic parameters after vehicle or phenylephrine administration and post second atrial pacing. **(E,H)** Hemodynamic parameters during second atrial pacing and after vehicle or phenylephrine administration.

**FIGURE 4:**
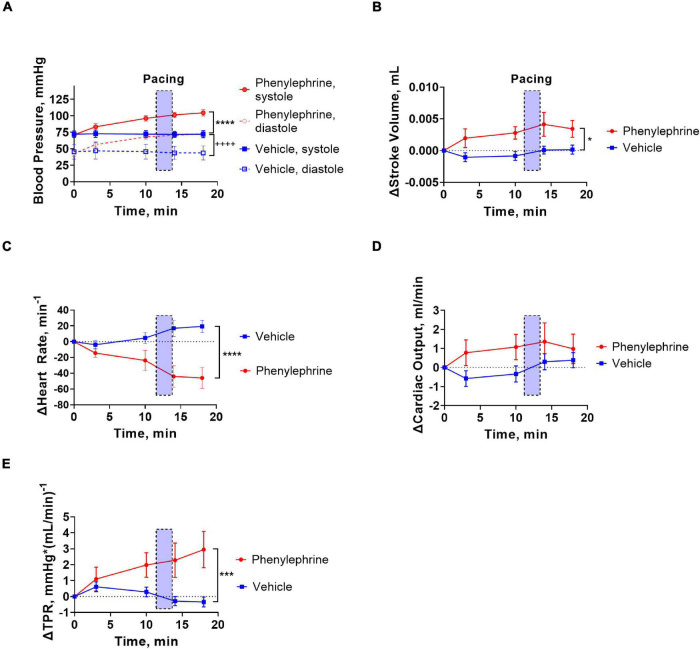
Vehicle or phenylephrine injection was given at the beginning of the traces (*x* = 0). Transparent blue boxes indicate the second pacing session corresponding to the panels in Figures 3E,H. **(A)** Following intervention with phenylephrine or vehicle, both systolic and diastolic blood pressures were significantly elevated in the phenylephrine group (****P < 0.0001 systolic pressure and ^++++^*P* < 0.0001 for diastolic pressure). **(B)** Stroke volume was also increased significantly following phenylephrine administration (*P = 0.027). **(C)** Heart rate was significantly decreased after phenylephrine administration in comparison with vehicle (****P < 0.0001). **(D)** Cardiac output was not changed significantly following phenylephrine administration (*P* = 0.21). **(E)** After phenylephrine administration, total peripheral resistance (TPR) significantly increased compared to vehicle group (***P = 0.0006). Data were compared with two-way ANOVA, *n* = 6.

As expected, following administration of phenylephrine, both systolic and diastolic blood pressures were significantly elevated (Figures 3, 4A). Stroke volume was also increased after administration of phenylephrine (Figure 4B) while heart rate was significantly reduced in comparison with the vehicle group (Figure 4C). Accordingly, cardiac output was not changed after phenylephrine administration and was not different from the vehicle group (Figure 4D). Total peripheral resistance was calculated as blood pressure divided by the corresponding values for cardiac output for the analyzed period. The calculated total peripheral resistance was significantly increased after phenylephrine administration (Figure 4E). Altogether, our data suggest that phenylephrine elevated blood pressure primarily due to an increased total peripheral resistance.

### Pacing Frequency Affects Cardiac Output but Not Total Peripheral Resistance

**FIGURE 5:**
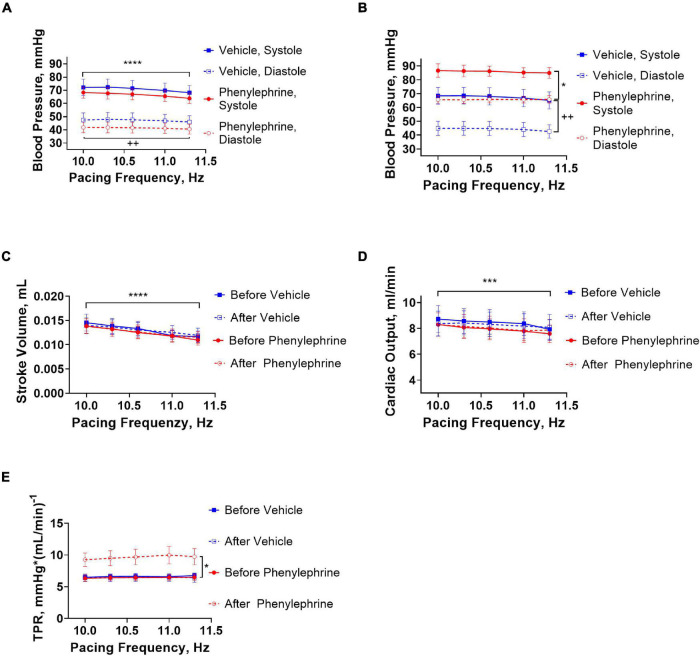
**(A)** During the first pacing session, prior to administration of vehicle or phenylephrine, blood pressure was decreased with increasing pacing frequencies in both groups (****P < 0.0001 for systolic pressure and ^++^*P* = 0.0028). **(B)** During the second pacing session after administration of phenylephrine, blood pressure did not change with increasing pacing frequencies, but elevation of blood pressure compared to vehicle group was maintained (*P = 0.028 for systolic blood pressure and ^++^*P* = 0.0064 for diastolic pressure). **(C)** Stroke volume decreased significantly with increasing pacing frequencies (****P < 0.0001). **(D)** Cardiac output was also affected with increasing pacing frequencies (***P = 0.0002). **(E)** Total peripheral resistance did not change with increasing pacing frequencies, but the phenylephrine-induced increments in total peripheral resistance were maintained during the second pacing session compared to the vehicle group (*P = 0.04). Data were compared with two-way ANOVA, *n* = 6.

The increasing pacing frequency from 10 to 11.3 Hz significantly reduced systolic and diastolic blood pressures under control conditions in both the phenylephrine and vehicle groups (Figure 5A). In contrary, increasing pacing frequencies had no significant effect on systolic and diastolic blood pressures during the second pacing session for the vehicle or phenylephrine groups (Figure 5B). Stroke volume and cardiac output was significantly reduced with increasing pacing frequency both under control conditions and following phenylephrine and vehicle administration (Figures 5C,D). We did not observe changes in total peripheral resistance with increasing pacing frequencies, neither during control conditions or following phenylephrine and vehicle administration (Figure 5E). Moreover, the increased blood pressure and total peripheral resistance following phenylephrine administration compared to the vehicle group remained unaltered during the second pacing session (Figures 5B,E).

## Discussion

This study reports on a mouse model for the assessment of cardiovascular parameters upon *in vivo* experimental interventions in a comprehensive and integrated manner. This model allows for distinction between interventions that have primary cardiac or vascular actions. We used α_1_ adrenoceptor agonist stimulation to modulate vascular function and observed the subsequent effects on overall cardiovascular function. By assessing the response to increasing pacing frequencies compared to periods of rest both with and without phenylephrine, we were able to differentiate between cardiac and vascular effects. We reported complex changes in blood pressure, heart rate, cardiac output, and total peripheral resistance following the interventions.

We chose a selective α_1_-adrenergic receptor agonist, phenylephrine, as it is known to increase blood pressure *via* elevation of systemic vascular resistance without any direct effect on myocardial contractility (Thiele et al., 2011). Phenylephrine is often used clinically as a vasopressor to treat arterial hypotension induced by general anesthesia (Kalmar et al., 2018). Moreover, phenylephrine is broadly used in *ex vivo* organ bath assessments of vascular functions (Gutierrez et al., 2019), but also *in vivo* in rodent experimental models (Boguslavskyi et al., 2021; Ralph et al., 2021) where it is used to provoke blood pressure changes. The α_1_-adrenergic mechanism underlying the vasopressor effect of phenylephrine is well-described (Dessy et al., 1998; Jumrussirikul et al., 1998; Brozovich et al., 2016; Boguslavskyi et al., 2021). Accordingly, we suggest that the significant phenylephrine-induced elevation of blood pressure in this study is a result of vascular constriction with consequent increase in total peripheral resistance. Furthermore, these elevations in blood pressure and total peripheral resistance were accompanied by a decrease in heart rate. We attributed this heart rate change to be a response to increased activation of baroreceptors, i.e., baroreflex. Upon blood pressure induced activation, the baroreceptors, mediated through the activity of the autonomic nervous system, exercise changes to both cardiac and vascular functions, thus, counteracting any acute pressure changes (Andresen and Kunze, 1994; Jumrussirikul et al., 1998; Boguslavskyi et al., 2021). In our experiments, the baroreceptors failed to return the blood pressure to resting values following phenylephrine injection. This is most likely because phenylephrine-mediated α_1_-adrenergic activation of vascular smooth muscle cells bypassed the baroreceptor-mediated inhibition of sympathetic outflow. As a result, phenylephrine maintained vasoconstriction increasing total peripheral resistance and blood pressure. These phenylephrine-induced changes in blood pressure, total peripheral resistance, and heart rate are also described in humans (Crosley et al., 1951; Malhotra et al., 1998).

Phenylephrine administration increased stroke volume. We suggest that this may be result of both increased preload because of phenylephrine-induced venous constriction (Jumrussirikul et al., 1998; Kalmar et al., 2018; Boguslavskyi et al., 2021) and a baroreceptor mediated reduction in heart rate with subsequent increased diastolic filling time. However, this stroke volume elevation is limited by simultaneous elevation of afterload due to increased total peripheral resistance as described previously (Yang et al., 2013; Kalmar et al., 2018). As a product of stroke volume and heart rate, cardiac output did not change significantly. Studies in humans reported both decreased and increased cardiac output following phenylephrine administration because of either increased afterload or reduced heart rate and an increased preload, respectively (Mon et al., 2017; Kalmar et al., 2018). A previous study in pigs suggested this bi-directional effect of phenylephrine to be related to changes to preload and, thus, preload level (Cannesson et al., 2012). Hence, in situations with significantly reduced end-diastolic ventricular filling, e.g., hemorrhage and hypotension, phenylephrine increases preload with consequent elevation in stroke volume and cardiac output (Cannesson et al., 2012). This is supported by observations of increased cardiac output following phenylephrine administration to hypotensive patients (Kalmar et al., 2018). This effect of phenylephrine is predominantly mediated by venous constriction and elevation of venous return leading to increased preload and stroke volume. The lack of a significant increase of cardiac output in this study may suggest that these mice were under relatively mild hypotensive conditions, or that the relation between preload-dependence and cardiac output in mice is different in comparison with humans and pigs (Georgakopoulos and Kass, 2001; Cannesson et al., 2012; Mon et al., 2017; Kalmar et al., 2018).

Mice have high resting heart rates ranging from 500 to 700 beats per minute depending on the time of day (Kaese and Verheule, 2012) corresponding to a very short cardiac cycle length of 80–110 ms. Thus, minimal changes in heart rate can have significant consequences for diastolic duration, diastolic filling time, and stroke volume. The importance of increased ventricular filling time for stroke volume in mice is debated (Georgakopoulos and Kass, 2001). In our study, stroke volume and cardiac output decreased significantly with increasing pacing frequencies suggesting that the ventricular filling time is indeed important. Additionally, the observed difference in stroke volume between the phenylephrine and vehicle groups was abolished during electrical pacing. This implies the importance of reduced heart rate and increase in diastolic filling time for the phenylephrine-induced increments in stroke volume in mice.

A load-dependent biphasic force-frequency relation has previously been described for the murine heart with a positive relation at 400–600 beats per minute (bpm) and a negative force-frequency relation above 600 bpm (Georgakopoulos and Kass, 2001). Accordingly, we saw a negative force-frequency relation during the first pacing session when we paced the hearts with increasing pacing frequencies starting from 10 Hz that corresponds to 600 bpm (Supplementary Figure 1). However, the force-frequency relations were significantly changed for the vehicle and phenylephrine groups during the second pacing session (Supplementary Figure 1). This change may be due to volume loss leading to compensatory endogenous catecholamine release from the sympathetic nerves and adrenal medulla with consequent increase in venous return (Funk et al., 2013). Thus, alterations in preload may have changed the force-frequency relation in our model. Normalizing the murine biphasic force-frequency relation to preload has been suggested to flatten the force-frequency relation curve at high frequencies (Georgakopoulos and Kass, 2001). This may in part explain the decrease in blood pressure seen in both vehicle and phenylephrine groups during the first pacing session and the diminished effect of increasing pacing frequency during the second pacing session. The component of venous return may have been greater following phenylephrine administration compared to vehicle when taking into account phenylephrine-induced venous constriction (Cannesson et al., 2012). Further measurements of pressure-volume relations in the caval vein or right atrium would answer this question.

The versatility of this model should be stated. For instance, this *in vivo* model can further be elaborated to include myocardial ischemia and reperfusion with temporary occlusion of a coronary artery and, thus, the systemic effects of acute myocardial infarction can be investigated (Michael et al., 1995). Additionally, the protocol may also be applied to other animal models, e.g., the rat model. However, considerations must be taken in regards of different electrophysiological properties of the heart beckoning other settings for the electrical pacing. This remains to be validated in the specific animal models.

Our protocol can distinguish between vascular and cardiac changes and possibly answer important questions in cardiovascular research. This can be particularly pertinent to disease states with multimorbid cardiovascular pathology. For example, cardiovascular morbidity in diabetes and hypertension can comprise pathological changes to both cardiac and vascular functions and lead to heart failure with preserved ejection fraction (Borlaug, 2020). Differentiating between the intrinsic cardiac properties and vascular function may shed light on the mechanism underlying this type of heart failure potentially improving treatment strategies. Currently, this can be examined by a combination of *in vivo* and *ex vivo* models, while our protocol offers a purely *in vivo* setting (Pedersen et al., 2018). Another important issue in cardiovascular research relates to molecules, e.g., ketone bodies and cardiac glycosides, that exert complex responses in the body (Tisdale and Gheorghiade, 1992; Nielsen et al., 2019). Ketone bodies have been described to increase cardiac output (Nielsen et al., 2019). However, due to possible multifarious effects on the cardiovascular system, it is unclear whether this increase in cardiac output is (i) due to increase in contractility or a decrease in heart rate, i.e., a direct cardiac effect, (ii) due to reduced total peripheral resistance, i.e., a direct vascular effect, or (iii) due to a combination of both vascular and cardiac effects. Thus, our model may be a helpful approach to illuminate such intricate research questions.

## Limitations

This experimental protocol provides a possibility to assess valuable comprehensive information on the cardiovascular system, but some limitations must be highlighted and considered for future elaboration of the model. The limited translational value of mouse studies must be considered (Perlman, 2016), yet, mouse experimental models are indispensable in mechanistic studies with genetic expression manipulations (Chinwalla et al., 2002) as well as for initial pharmacological trials and other high throughput analyses (Perlman, 2016). Presently, multiple animal studies must be performed to gain comprehensive knowledge on the effects of a pharmacological treatment. In this regard, our protocol is helpful in reducing the number of animal experiments needed for the comprehensive assessment of *in vivo* pharmacological interventions in the cardiovascular system.

The established protocol is technically challenging as it is an invasive procedure and requires the introduction of several probes. In this regard, smaller animals may yield a lower surgical success rate due to lower surgical accessibility and increased vulnerability compared to bigger animals. This demands higher surgical skill level and precision. In our experiments, we used 9 months old mice which had suitable size for the surgery required.

Several other aspects related to the surgical procedure must also be mentioned. Opening the thorax alters pressure dynamics in the thoracic cave, e.g., pressure alterations related to natural respiration are eliminated, and this may affect venous return and cardiac function. Additionally, anesthesia and open-thorax interventions have previously been reported to lead to a significant drop in blood pressure (Hoit et al., 1997; Janssen et al., 2004). To reduce the significance of mechanical ventilation between the vehicle and phenylephrine groups, the ventilation parameters were maintained throughout the entire experiment and were comparable between the two groups. In addition, parameters such as positive end-expiratory pressure, airway pressure, and flow were monitored and kept constant in all experiments. Partially, these factors are probably also the reason for the slight hypotensive condition during the experiment. The hypotensive conditions can be corrected by vasopressor administration, e.g., phenylephrine. However, considerations must be taken for the possible interference of several cardiovascular parameters including a direct effect on peripheral vascular resistance with indirect effects on cardiac function as has been shown in this study. Lastly, pancuronium bromide was used in this open-thorax protocol as a muscle relaxant to eliminate intrinsic respiratory reflexes. Pancuronium has been reported to increase heart rate and contractility in rat atrial preparations (Gursoy et al., 2011). Similarly, heart rate but also blood pressure has been described to increase following administration of pancuronium in anesthetized patients (Pauca and Skovsted, 1981). However, we did not see any significant cardiovascular changes in response to intraperitoneal injection of pancuronium used in our study.

Overdriving of heart rate with electrical pacing is not a normal physiological condition. Electrical pacing of the heart with increasing frequency is commonly used in arrhythmia research, e.g., programmed electrical stimulation protocols. However, the pacing settings in our study did not modify the electrical properties of the heart nor introduce arrhythmias evident from ECGs. Electrical pacing successfully and consistently controlled the heart rhythm in the frequency range of 10–11.3 Hz corresponding to a physiological heart rate of 600–678 BPM. Pacing with frequencies below 10 Hz or above 11.3 Hz did not always translate into a 1:1 capture of the heart rhythm. Moreover, recovery of hemodynamic parameters after electrical pacing indicates that cardiovascular functions were not damaged compared to their normal physiological state. Lastly, our results suggest that the electrical pacing had no direct effect on vascular function.

We measured blood flow in the ascending aorta as surrogate for cardiac output. This did not include the blood flow through the coronary arteries and, thus, underestimates the true value of cardiac output. Moreover, change in venous return is an important parameter in our mechanistic assessment, yet, this was not measured directly as this will further complicate the setup. These improvements, e.g., venous blood flow or an atrial pressure-volume assessment, would be an advantage for future technical development. Lastly, despite the importance of neurohumoral regulation for the cardiovascular system, this was not assessed in this study, but this can be considered in future research.

## Conclusion

In conclusion, this *in vivo* protocol provides a method of controlling heart rate in cardiovascular studies in the murine model with pharmacological intervention. Phenylephrine administration increased total peripheral resistance, blood pressure, and stroke volume. However, during atrial pacing there were no differences in stroke volume and cardiac output between the phenylephrine and vehicle groups. Thus, our model suggests that the decrease in heart rate with consequent increase in ventricular filling may be a significant contributor to the phenylephrine-induced increase in stroke volume rather than increase in venous return. This model underlines the primary vascular effects of phenylephrine with secondary cardiac changes. Although technically challenging, this protocol enables the assessment of a broad range of parameters for an integrated understanding of the cardiovascular system under normal and pathological conditions, and it may be helpful in answering important questions in cardiovascular physiology.

## Data Availability Statement

The animal study was reviewed and approved by the raw data supporting the conclusions of this article will be made available by the authors, without undue reservation.

## Ethics Statement

The experimental protocol was approved by the Animal Experiments Inspectorate of the Danish Ministry of Environment and Food and reported in accordance with the ARRIVE (Animal Research: Reporting *in vivo* Experiments) guidelines.

## Author Contributions

RR and VM performed the experiments and made the draft of the manuscript. RR, TP, and VM performed the data analysis. RR, TP, MT, and VM took part in the conception and designed the study. All authors provided critical feedback and approved the final version of the manuscript.

## Conflict of Interest

The authors declare that the research was conducted in the absence of any commercial or financial relationships that could be construed as a potential conflict of interest.

## Publisher’s Note

All claims expressed in this article are solely those of the authors and do not necessarily represent those of their affiliated organizations, or those of the publisher, the editors and the reviewers. Any product that may be evaluated in this article, or claim that may be made by its manufacturer, is not guaranteed or endorsed by the publisher.
