# Supplementary material for: Phenylephrine-Induced Cardiovascular Changes in the Anesthetized Mouse: An Integrated Assessment of in vivo Hemodynamics Under Conditions of Controlled Heart Rate
Source: Front Physiol. 2022 Feb 17;13:831724. doi: 10.3389/fphys.2022.831724 (PMC8891648; doi:10.3389/fphys.2022.831724)

Supplementary Figure 1. Differences in the maximum slope of the blood pressure profiles during systole between the first and second pacing sessions for both the vehicle (**P* = 0.016) and phenylephrine (^{++}P = 0.0013) groups were observed. Data were compared with two-way ANOVA, n = 6.

Supplementary Figure 1

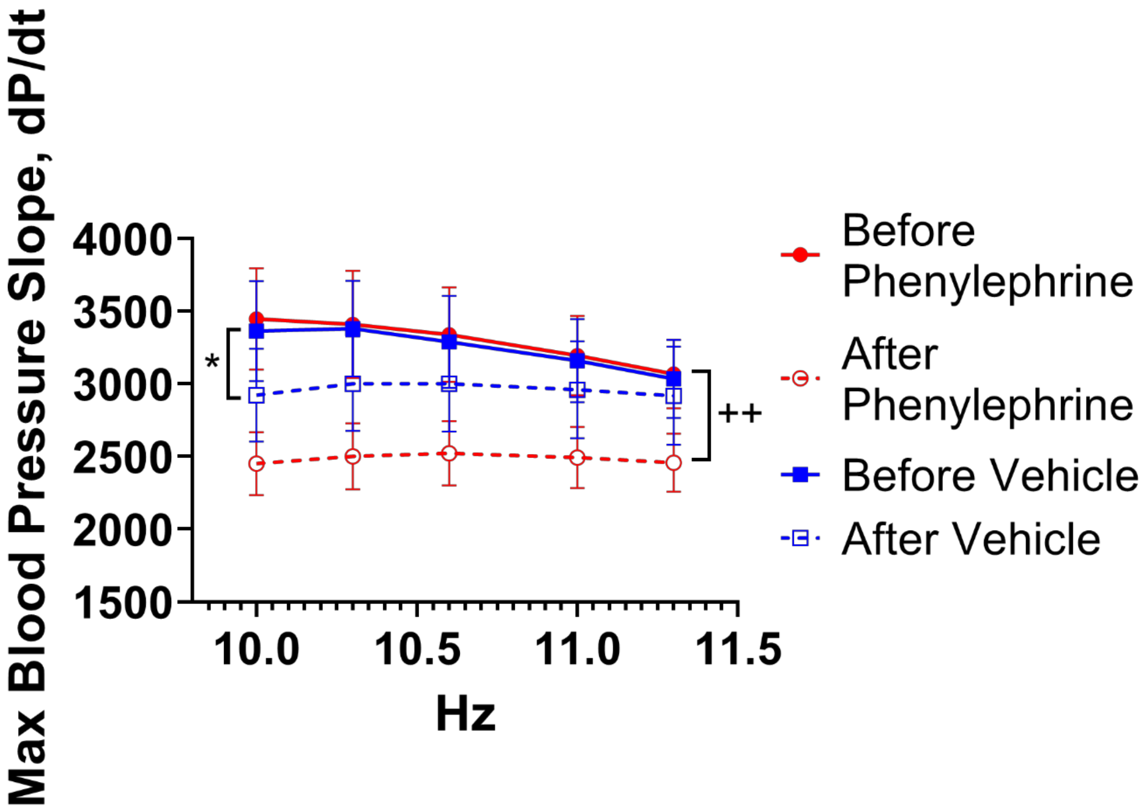

Supplement: Supplementary file 1 [file Image_1.pdf]
